# Beyond VI-RADS Uncertainty: Leveraging Spatiotemporal DCE-MRI to Predict Bladder Cancer Muscle Invasion

**DOI:** 10.3390/bioengineering12121338

**Published:** 2025-12-08

**Authors:** Minghui Song, Haonan Ren, Lijuan Wang, Yihang Zhou, Xing Tang, Huanjun Wang, Yan Guo, Yang Liu, Hongbing Lu, Xiaopan Xu

**Affiliations:** 1 School of Biomedical Engineering, Air Force Medical University, Xi’an 710032, Chinazhouyihang200304@163.com (Y.Z.);; 2 School of Information and Communication Engineering, Hainan University, Haikou 570228, China; 3 School of Life Science and Technology, Xi’an Jiaotong University, Xi’an 710049, China; 4 Department of Radiology, Xijing Hospital, Air Force Medical University, Xi’an 710032, China; tangxing1984@163.com; 5 Department of Radiology, The First Affiliated Hospital of Sun Yat-Sen University, Guangzhou 510080, China

**Keywords:** multi-temporal-phase DCE-MRI, non-muscle-invasive bladder cancer, VI-RADS, TM3DConvNet, multiscale spatiotemporal information

## Abstract

**Background**: The Vesical Imaging-Reporting and Data System (VI-RADS) has limited diagnostic accuracy in distinguishing non-muscle-invasive bladder cancer (NMIBC) within VI-RADS categories 2 and 3, despite its value for overall NMIBC assessment. Dynamic contrast-enhanced MRI (DCE-MRI), which reflects tumor vascularity, holds promise for improving these challenging cases but remains underutilized due to unexploited spatiotemporal information. **Methods:** We developed a deep learning model to comprehensively quantify spatiotemporal features from multiphase DCE-MRI in 184 patients with VI-RADS 2 or 3 (training: *n* = 115, validation: *n* = 20, testing: *n* = 49). The model integrated multiscale feature extraction and contextual attention mechanisms to enhance diagnostic performance. **Results:** The model outperformed established benchmarks (e.g., VGG, ResNet) and the conventional VI-RADS ≤ 2 threshold (sensitivity: 0.67 for NMIBC), achieving a sensitivity of 0.90 (95% CI: 0.81–0.96) for NMIBC and an area under the curve (AUC) of 0.82 (95% CI: 0.75–0.89) for overall classification. Visualizations confirmed its ability to identify key spatiotemporal patterns linked to muscle invasion. **Conclusions:** By leveraging comprehensive spatiotemporal information from DCE-MRI, our deep learning model significantly improves NMIBC diagnosis in VI-RADS 2/3 cases, offering a clinically valuable tool to address the limitations of current VI-RADS assessment.

## 1. Introduction

Urinary bladder cancer (BCa) is the fourth most common malignancy in male patients globally [1,2]. Among initial diagnoses, approximately 75% are classified as non-muscle-invasive bladder cancers (NMIBCs) [3,4], while the remaining 25% are muscle-invasive bladder cancers (MIBCs) [5]. Treatment strategies for NMIBCs focus on preventing progression and ensuring close surveillance for muscularis invasion [3,4]. In contrast, MIBC often necessitates radical cystectomy to mitigate lymph node involvement and prevent distant metastasis. Accurate pretreatment diagnosis of NMIBC is crucial for developing personalized treatment and management of patients.

The reference standard for diagnosing NMIBC is transurethral resection of bladder tumor (TURBT) followed by pathological examinations [3,5]. Due to the alteration of tissue architecture resulting from TURBT or the absence of detrusor muscle in the resected specimens, approximately 39% of BCa cases are at risk of being under-staged [4]. Repeated TURBTs may help reduce the diagnostic error, but are invasive, uncomfortable, time-consuming, and costly [4,6,7].

Recent advancements in MRI have established multiparametric MRI (mp-MRI) as a valuable complement for the preoperative diagnosis of detrusor muscle infiltration of BCa [8,9]. Mp-MRI consists of structural sequences, such as T2-weighted imaging (T2WI), and functional sequences, including diffusion-weighted imaging (DWI) and multiphase dynamic contrast-enhanced MRI (DCE-MRI) [8]. These MRI sequences jointly provide excellent contrast resolution for BCa staging, early recurrence detection, and treatment response assessment [8,9]. To standardize mp-MRI scanning and reporting for BCa, the Vesical Imaging-Reporting and Data System (VI-RADS) was introduced in 2018 [8,9,10,11], which employs a five-point scoring system to assess the likelihood of detrusor muscle infiltration [8,10].

According to the VI-RADS scoring system, a score of 1 indicates a high probability of non-muscle invasive bladder cancer (NMIBC), while scores of 4 or 5 suggest strong evidence of muscularis invasion [10,12,13]. Recent studies have shown that between 4.1% and 17.4% of patients with a score of 2 were later diagnosed with MIBC after surgery [6]. Additionally, a score of 3 presents significant challenges in precisely distinguishing between NMIBC and MIBC [7]. This issue has resulted in some patients with VI-RADS scores of 2 or 3 being incorrectly staged, especially when the common threshold of VI-RADS ≤ 2 is used to predict NMIBC [13].

To deal with these issues, Cai et al. developed an artificial intelligent (AI) model to predict NMIBC in VI-RADS score of 2 with tumor stalk [6], and Yu et al. proposed another AI model to predict the muscularis invasion among patients with VI-RADS score of 3 [7]. To the best of our knowledge, few AI models have been specifically developed for the accurate prediction of muscularis invasion in patients with VI-RADS 2 or 3.

When investigating all the sequences in the mp-MRI and VI-RADS scoring system, DCE-MRI employs gadolinium-based contrast agents with multiphase MRI scanning to reflect the dynamics of vascular permeability within the tumor and its surrounding bladder wall, which potentially provides important dynamic spatiotemporal information for NMIBC prediction [14,15]. However, most existing studies have concentrated primarily on the peak enhancement phase post-contrast administration [2,16,17,18,19], often overlooking the rich spatiotemporal information that can be gleaned from the dynamic perfusion process during multi-temporal-phase DCE-MRI scans [20].

Currently, deep learning (DL) models such as Visual Geometry Group-16 (VGG-16), ResNet-101, DenseNet-121, and Vision Transformer (ViT) have gained great attention in the field of image-based cancer diagnostics [9,21,22,23,24,25]. However, these models often face challenges in effectively capturing the dynamic spatiotemporal information in the multi-temporal-phase DCE-MRI. A recent study by Bedel et al. introduced a BOLD transformer model that uses a series of transformer encoders combined with a new fused window attention mechanism to capture spatiotemporal information from time-series functional MRI [25], which provides valuable insights for analyzing multi-temporal-phase DCE-MRI in diagnosing NMIBC. Considering the significant variation in BCa masses among patients, a multiscale convolutional mechanism may enhance the capability of the AI model to capture both coarse and fine-grained image information [26,27], potentially improving the performance for NMIBC prediction [26,27].

Building upon these insights, the present study aimed to develop and validate a Transformer-fused multiscale 3D convolutional network (TM3DConvNet) that fully captures the multiscale spatiotemporal information from multi-temporal-phase DCE-MRI to improve NMIBC prediction among patients with VI-RADS scores of 2 or 3.

## 2. Materials and Methods

### 2.1. Patient Population

Datasets were collected by a senior radiologist (H.W., 15 years of experience) from the picture archiving and communication system at The First Affiliated Hospital of Sun Yat-Sen University. Institutional Review Board (IRB) approval was obtained (Approval No. [2020]492). The ethical approval date was 8 December 2020. These data included consecutive BCa patients who underwent preoperative mp-MRI scans between March 2013 and May 2018 and had surgical procedures within two weeks of their initial imaging exams. Tumors were analyzed pathologically, and for patients with multiple lesions, the tumor site with the highest burden (largest volume or most advanced stage) was selected for analysis [8,9].

The inclusion criteria were as follows: (a) patients suspected of primary BCa with axial mp-MRI scans and VI-RADS scores of 2 or 3 before treatment, (b) available pathological results within two weeks, and (c) at least five phases of axial DCE-MRI scans. Patients were excluded if they met any of the following conditions: (a) absence of VI-RADS scores of 2 or 3, surgical treatment records, or pathological findings, (b) merely sagittal or coronal MRI scans, (c) fewer than five phases axial DCE-MRI scans, and (d) severe artifacts or poor image quality. After applying these criteria, eligible patients were randomly assigned to the training, validation, and testing cohorts roughly at a 6:1:3 ratio, as shown in Figure S1.

Based on the criteria, 184 patients were eligible. Among them, 134 and 108 patients were previously reported in two separate studies [8,9]. The current study investigated whether multiscale spatiotemporal information from multi-temporal-phase DCE-MRI could effectively predict NMIBC, distinct from the previous studies focusing on recurrence risk assessment [8,9].

### 2.2. MRI Examination

Patients were positioned supine in the MRI unit after fasting for four to six hours and instructed to drink 500–1000 mL of water 30 min prior to ensure adequate bladder distension. MRI scans were conducted using a 3 Tesla system (Magnetom Trio, Siemens (Munich, Germany); Signa Pioneer, GE Healthcare (Chicago, IL, USA)) with an 8- or 32-channel body coil, covering the pelvic region from the aortic bifurcation to the lower edge of the pubic symphysis. The sequences included axial T2WI without fat suppression, high-spatial-resolution fat-suppressed T2WI, and axial DWI (b values of 0 and 1000 s/mm^2^ for Magnetom Trio, or 50 and 1000 s/mm^2^ for Signa Pioneer) [8,10]. Multi-temporal-phase DCE-MRI was conducted using a fat-suppressed volumetric spoiled gradient-echo sequence, both before and after intravenous injection of 0.2 mL/kg of body weight of gadopentetate dimeglumine or gadoteric acid at 2 mL/s [28,29]. Most patients underwent five phases of scanning within 20 to 131 s after contrast administration. The main scanning parameters are detailed in Table S1. All patients underwent a standardized 5-time-point DCE-MRI protocol (acquired at 0 s, 30 s, 60 s, 90 s, and 5 min post-contrast). However, due to patient individual differences (such as injection delay, preparation time, or equipment adjustments), the actual arrival time of the contrast agent at the target organ and the acquisition timing of corresponding phases varied significantly among individuals, resulting in differences in the selected acquisition time points. Minor variations in the actual number of acquired time-points occurred due to equipment constraints (60 s time-point omitted in 5% of cases) or patient-specific factors (e.g., reduced time-points in one case due to poor tolerance). The model’s input layer dynamically accommodated varying time-point numbers via multiscale convolution (e.g., adaptive channel adjustment using 1 × 1 convolutions), ensuring robust analysis. Definitions and acquisition timings for multi-temporal-phase DCE-MRI are detailed in Table S2.

### 2.3. Management of MRI Data Heterogeneity Arising from Coil Characteristics

The coil type (8-channel for smaller patients, 32-channel for larger patients/higher SNR needs) was chosen based on body habitus and scan region, and not randomly. All coils were body coils optimized for pelvic MRI (aortic bifurcation to pubic symphysis), sharing similar design and key features (coverage, field uniformity). To address the smooth, low-frequency intensity nonuniformity caused by imperfections in coil sensitivity and B1 field inhomogeneity, N4 bias field correction algorithm was implemented with its default parameter settings. In addition, to ensure the inter-subject and inter-scanner consistency, global intensity normalization was applied. Low-quality images (artifacts, signal loss, abnormal contrast) from coil faults, patient motion, or parameter errors were excluded during preprocessing. The final 184-patient dataset met strict criteria (“no obvious coil artifacts, high clarity, good tumor contrast”), further minimizing coil-related impacts.

### 2.4. Image Preassessment

The mp-MRI data for each patient were scored using a five-level scoring system at a two-week interval. Two senior radiologists (H.W and Y.G., with 16 and 36 years of experience, respectively) independently scored the images without prior knowledge of the patients’ surgical or histopathological findings. After scoring all MRI sequences, a VI-RADS score was assigned to each patient in accordance with established VI-RADS principles [10], and any discrepancies between the radiologists were resolved through consensus. Afterward, the senior radiologist (X.T., 15 years of experience) assessed the multi-temporal-phase DCE-MRI scans for each patient, identifying the tumor location in the five phases based on postoperative pathology results. For patients with more than five phases of data, only the pre-contrast and four following contrast phases were considered for further analysis.

### 2.5. Development of the Proposed TM3DConvNet Model

**Figure 1:**
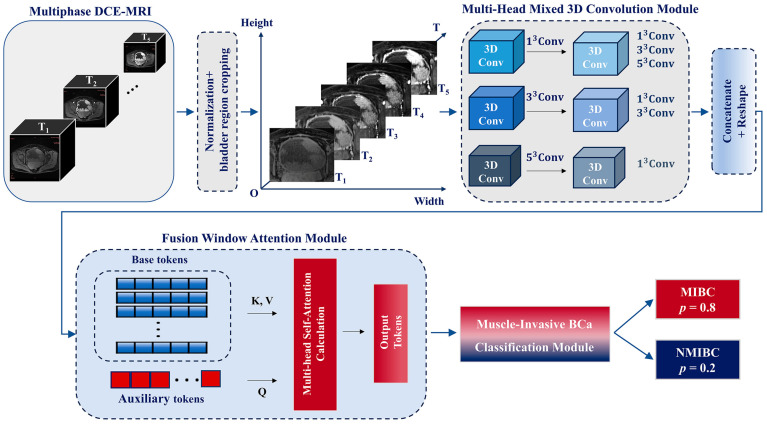
Overall methodological scheme of the proposed method.

The overall workflow of the proposed TM3DConvNet model is shown in Figure 1. Following image preassessment, the image slice with the largest tumor burden at that location of each phase was then selected, constituting a 3D multi-temporal-phase DCE-MRI data X ($X\in R^{H\times W\times 5}$) for further analysis.

#### 2.5.1. Normalization, ROI Cropping Block and Data Augmentation

To enhance the comparability of tumor signal intensity across multiple phases in dynamic contrast-enhanced magnetic resonance imaging (DCE-MRI), this study employed a Z-score normalization method based on region-of-interest (ROI) statistics to standardize the image data. First, a radiologist with 13 years of diagnostic experience (X.T.) manually delineated the bladder region containing the tumor on all temporal phase images to precisely define the region of interest (ROI). Subsequently, the raw signal intensity values of all voxels within the tumor region across all phases were extracted, and their overall mean (*μ*) and standard deviation (*σ*) were calculated. Based on these global statistical measures, Z-score normalization was performed on the signal intensity *S_raw_* of each voxel within the tumor region for each phase as follows:

$$S_{norm}\;=\;\frac{S_{raw}-\mu }{\sigma }$$

*S_raw_*: The unnormalized signal intensity (raw intensity value) in the original image.

*μ*: The mean signal intensity of all voxels within the tumor region across all temporal phases (global mean of training dataset).

*σ*: The standard deviation of signal intensity of all voxels within the tumor region across all temporal phases (global standard deviation of training dataset).

*S_norm_*: The normalized (standardized) signal intensity value after Z-score transformation.

Data augmentation was then performed using a conditional generative adversarial network to expand the minority group (MIBC samples), improving class balance for model construction [30] (refer to the Supplementary Materials). In addition, to simulate real-world noise variations, MRI-specific Rician noise and Gaussian noise were incorporated into the images with a defined probability. After that, common data augmentation techniques such as rotations, mosaic augmentation, scaling, and translations were further utilized with both groups of samples in the training cohort to improve the data diversity for model building.

#### 2.5.2. 3D Multi-Head Mixed Convolution (MHMC) Module

**Figure 2:**
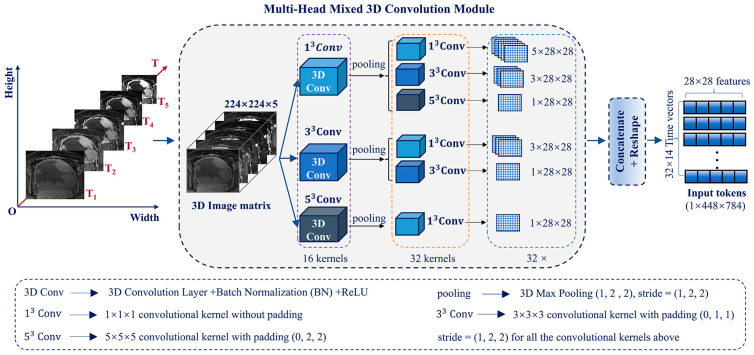
Framework of the multi-head mixed 3D convolution (MHMC) module.

Before performing multi-head convolution, the cropped 5-phase image slices were resized to match the dimensions of $X\prime (X\prime \in R^{224\times 224\times 5})$, as shown in Figure 2. An image size of 224 × 224 is widely adopted in many DL models. It ensures compatibility with these models while preserving sufficient resolution to capture detailed image features [27].

Subsequently, a lightweight 3D multi-head mixed convolution (MHMC) module was used to capture the multiscale spatiotemporal features of the 5-phase image slices. The MHMC module employed three parallel 3D convolution pathways with kernel sizes of 1 × 1 × 1, 3 × 3 × 3, and 5 × 5 × 5, which were designed to extract features at multiscale receptive fields, namely local, medium, and global spatial contexts, respectively.

The selection of multiscale convolutional kernels (1 × 1 × 1, 3 × 3 × 3, and 5 × 5 × 5) in our study was guided by the dual requirements of multiscale feature fusion and task adaptability: small kernels (1 × 1 × 1, 3 × 3 × 3) were specifically designed to capture localized, fine-grained tumor features, with their smaller receptive fields enabling precise delineation of critical microstructural details—including tumor margins, microvasculature, and textural heterogeneities—while minimizing information loss from over-smoothing; large kernels (5 × 5 × 5) were optimized to model global spatial characteristics of the tumor, as their extended receptive fields facilitate the integration of inter-regional feature correlations (e.g., contrasting intensely enhanced central zones with peripherally hypo-enhanced areas), effectively compensating for broader contextual information that smaller kernels might overlook. Given that the input consists of 5-phase 3D DCE-MRI sequences (each phase represented as a 3D volumetric dataset) requiring concurrent modeling of both spatial features (tumor morphology) and temporal features (dynamic enhancement kinetics), the multiscale kernel architecture (1 × 1 × 1, 3 × 3 × 3, 5 × 5 × 5) systematically addresses this multidimensional complexity by spanning a continuum of receptive fields—from single-voxel level detail (e.g., isolated enhancement disparities) to whole-tumor patterns (e.g., the 5-phase integrated enhancement signature)—thereby optimally accommodating the structural intricacies of 3D medical imaging data. Furthermore, although larger kernels (e.g., 7 × 7 × 7) could provide even more extensive receptive fields, we prioritized the combination of 1 × 1 × 1, 3 × 3 × 3, and 5 × 5 × 5 to achieve an optimized trade-off: this configuration preserves feature diversity across spatial scales, enhances model generalization in small-sample scenarios by mitigating overfitting risks, and maintains computational efficiency, which are key considerations for clinical translation. Each pathway consisted of a dual-layer convolutional structure: the first layer applied a standard 3D convolution with kernel w_a_, followed by batch normalization, rectified linear unit (ReLU) activation, and max pooling; the second layer utilized depth-wise separable convolutions (comprising a depth-wise convolution followed by a 1 × 1 × 1 pointwise convolution) to further refine features while significantly reducing the computational cost. This two-stage design enabled efficient yet expressive extraction of multiscale information from the input 5-phase DCE-MRI slices $X\in R^{H\times W\times 5}$.

Formally, the feature extraction process in each branch can be expressed as:

(1) $${X_{i}}^{\prime \prime }=\;reshape\left(w_{b}\left(w_{a}X\right)\right),\;i=1,3,5$$

where X is the input 5-phase image slice with dimensions *H × W × 5*, $w_{a}$ and $w_{b}$ are learnable convolutional kernels in the dual-layer structure, and reshape (⋅) is an operation that transforms the feature dimensions from 3D to 2D for further processing.

After feature extraction across the three parallel branches, the resulting multi-scale feature maps were concatenated along the temporal (or phase) dimension to form a unified token representation. Specifically, the outputs ${X_{1}}^{\prime \prime },{X_{3}}^{\prime \prime }\;$ and ${X_{5}}^{\prime \prime }$ from the three branches were combined as follows:

(2) $$X^{\prime \prime }=Concat({X_{1}}^{\prime \prime },{X_{3}}^{\prime \prime },{X_{5}}^{\prime \prime })$$

The final output token $X^{\prime \prime }$ has the shape 1 × 448 × 784 (e.g., for one dataset instance: 1 sample, 448 time points or spatial elements, and 784 features per time point), effectively encapsulating multiscale spatial and contextual information from all five DCE-MRI phases. This compact yet informative representation serves as the input to the subsequent fused window attention module for higher-level spatiotemporal modeling.

#### 2.5.3. Fusion Window Attention Module (FWAM)

**Figure 3:**
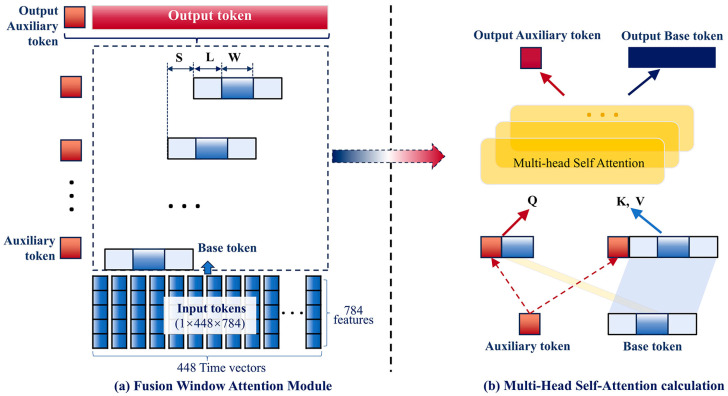
Illustration of the multiscale spatiotemporal attention capture and information fusion: (**a**) Transformer-based fusion window attention module (FWAM); (**b**) the multi-head self-attention calculation within the window.

The original self-attention mechanism effectively calculates global correlations in the input token, proving successful in natural language processing and computer vision. However, it struggles with larger input dimensions and increased computational complexity, often overlooking the fine-grained correlations within parts of the input token [25,27]. To address these issues, we introduced the FWAM (Figure 3).

The FWAM adopted Transformer blocks to split each input token $X^{\prime \prime }$ into windows of W time points, with a stride of S time points between adjacent windows [25]. Each window includes a fringe of L time points on both sides, forming a base token of dimensions 1+(L+W+L)×784, as illustrated in Figure 3a. The corresponding formula is:

(3) F=(T−W)/S+1

where T is the total 448 time points in this framework; W represents the size of the time points in each window, and the stride between adjacent windows is S. From this formula, the total number of token windows F to be fused can be determined.

An auxiliary token, initialized as a shared, learnable vector, captured high-level features for NMIBC prediction by summarizing the 448 time points. As the input token $X^{\prime \prime }$ passed through the Transformer blocks, the auxiliary token and base token of each window were used for multi-head self-attention calculations, as shown in Figure 3b. Specifically, the base token $x_{i}\in R^{W\times N}$ (representing the central Wtime points) and the auxiliary token(s) $a_{i},b_{i}\in R^{L\times N}$ (representing the left and right fringe time points) were concatenated with a context vector $C_{i}\in R^{1\times N}$ to form the query ($Q_{i}$), key ($K_{i}$), and value ($V_{i}$) vectors as follows:

(4) $$Q_{i}\;=W_{q}\{c_{i},x_{i}\}\;\in R^{(1+W)\times N}$$

(5) $$K_{i}=V_{i}=W_{k}\{c_{i},a_{i},x_{i},b_{i}\}\;\in R^{(1+W+2L)\times N}$$

where $W_{q}$ and $W_{k}$ are the learnable projection parameters, The self-attention scores were then computed using the scaled dot-product attention mechanism:

(6) $$attn(Q_{i},K_{i},V_{i})=\sigma (\frac{Q_{i}{K_{i}}^{T}}{\sqrt{d_{i}}})V_{i}$$

where $d_{i}$ is the dimension of input features, and used to stabilize the gradients during training, σ is softmax(⋅) function.

Each window had its own version of the auxiliary token to capture fine-grained attention. The output auxiliary tokens and output tokens were then fed into the classification module for NMIBC prediction.

After obtaining the final classification variable $c_{i}$, we aggregate the learned time features from all windows, weighted by their respective importance,

(7) $$F_{o}=\frac{1}{F}\sum_{i=1}^{F}c_{i}$$

where $F_{o}$ is the representative features after feature fusion. Subsequently, layer normalization was applied to stabilize the hidden states at each time point and mitigate the gradient explosion problem.

#### 2.5.4. Classification Module

After the FWAM, all output tokens were converted into an embedding vector which contained the aggregative spatiotemporal information. Then a fully connection layer was designed to predict the probability of label embedding $y_{p}$ for each subject [25]. Then, the cross-entropy loss function calculating the classification loss between ground truth label y and the prediction $y_{p}$ for each subject [25], was used to minimize the classification error. The probability $y_{p}$ was computed using a fully connected layer as:

(8) $$y_{p}=\sigma (W_{o}F_{o}+b_{o})$$

where $W_{o}$ and $b_{o}$ are the weight matrix and bias matrix, respectively, and σ(·) is the sigmoid function.

The cross-entropy loss function was defined as:

(9) $$L_{loss}=\;-\frac{1}{N}\sum_{i=1}^{N}\left[y\cdot \log\left(y_{p}\right)+(1-y)\cdot log(1-y_{p})\right]$$

### 2.6. Performance Evaluation and Comparison

The TM3DConvNet model was developed using the training cohort, with NMIBC cases labeled as negative. The model was then validated in the testing cohort and compared to common DL models like VGG-16, ResNet-101, DenseNet-121, and ViT for NMIBC diagnosis. Additionally, the performance using multi-temporal-phase DCE-MRI data was compared to the single-phase DCE-MRI for NMIBC diagnosis. All models were run on a lab server with Windows 10, an NVIDIA GeForce RTX 3090 GPU, 10,496 CUDA cores, and 24 GB of VRAM.

### 2.7. Statistical Analysis

Statistical analyses were conducted using SPSS software (version 20; IBM Corp., Armonk, NY, USA) and R software (version 4.1.1; R Foundation for Statistical Computing, Vienna, Austria). The interobserver agreement of the VI-RADS score was evaluated with the weighted k value. The performance of the proposed model and other common DL models was evaluated using sensitivity, specificity, accuracy, and area under the curve (AUC). DeLong’s tests with Bonferroni correction were used to compare the receiver operating characteristic (ROC) curves of these models for NMIBC diagnosis [31]. A two-tailed *p*-value < 0.05 was considered statistically significant.

## 3. Results

### 3.1. Demographics of Eligible Patients

An interobserver agreement analysis was conducted to assess the VI-RADS scoring consistency between two radiologists, yielding a weighted kappa value of 0.86 (95% CI: 0.78, 0.89). After that, 184 patients (median age 67 years, range 55–72; 15 women) were eligible. Among them: (i) 136 (73.9%) had NMIBC and 48 (26.1%) had MIBC; (ii) 141 (76.6%) had solitary tumors and 43 (23.4%) had two to four synchronous tumors; (iii) 121 (65.8%) underwent TURBT operation. Detail information is shown in Table 1.

### 3.2. Performance Comparison of the Proposed TM3DConvNet with Other Models

The prediction performance of the proposed TM3DConvNet model was comprehensively evaluated in both the training and testing cohorts, and systematically compared with widely used deep learning models, including VGG-16, ResNet-101, DenseNet-121 and ViT. In addition to deep learning models, we performed comparative analysis with the conventional imaging assessment method—the Versatile Integrated Radiological Assessment for Bladder Cancer Staging (VI-RADS). Two board-certified radiologists (H.W. and Y.G.) with extensive experience in urogenital imaging independently evaluated the multiphase DCE-MRI examinations according to standardized VI-RADS criteria (1–5 scale), where scores 1–2 correspond to non-muscle-invasive bladder cancer (NMIBC) and scores 3–5 indicate muscle-invasive bladder cancer (MIBC). To ensure diagnostic consistency, any scoring discrepancies were resolved through consensus discussion between the evaluating radiologists. The diagnostic performance of VI-RADS in differentiating NMIBC from MIBC was determined based on the final consensus scores and corresponding pathological findings, demonstrating a sensitivity of 70% (95% CI: 63–77), specificity of 80% (95% CI: 73–86), and overall accuracy of 0.73 (95% CI: 0.69–0.83).

**Figure 4:**
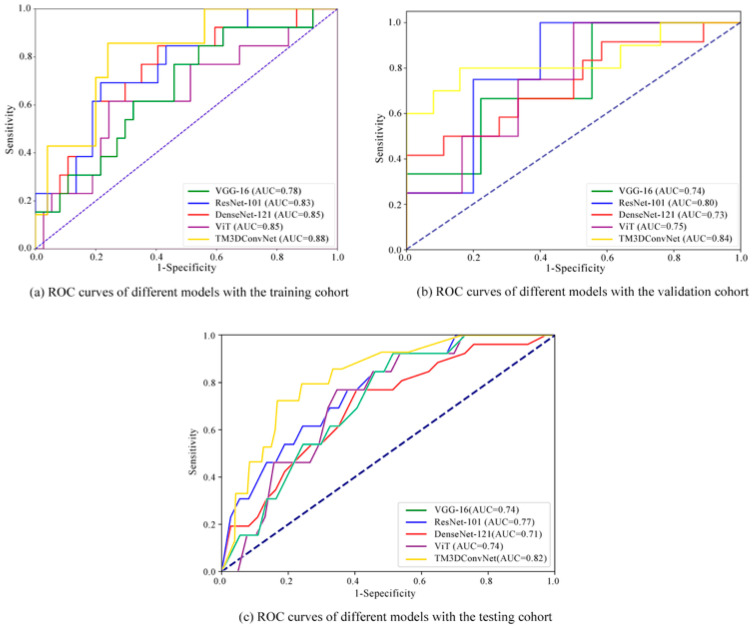
Comparison of TM3DConvNet with other widely-used DL models for NMIBC prediction using multi-temporal-phase DCE-MRI data: (**a**) receiver operating characteristic (ROC) curves on the training cohort, (**b**) ROC curves on the validation cohort, and (**c**) ROC curves on the testing cohort, respectively.

Figure S2 shows the training loss curves of these models, in which TM3DConvNet exhibited the fastest convergence speed and the lowest loss value. Figure 4 and Table 2 present the performance of TM3DConvNet, established benchmark networks (VGG, ResNet, DenseNet, Vision Transformer), and the conventional VI-RADS (≤2-score threshold) for NMIBC prediction across the training, validation, and testing cohorts. In the testing cohort, TM3DConvNet demonstrated superior performance, achieving a sensitivity of 0.90 for NMIBC and an area under the curve (AUC) of 0.82 for overall classification. Table 3 presents DeLong’s tests with Bonferroni correction to analyze the differences in the receiver operating characteristic (ROC) curves between TM3DConvNet and the established benchmark networks in the testing cohort. Table 4 shows the ablation experiments of the important modules designed in the proposed TM3DConvNet model for predicting bladder cancer detrusor muscularis invasion.

Additionally, the performance of the benchmark networks with single-phase DCE-MRI was also evaluated in the testing cohort, as shown in Table S2, which was inferior to that of the proposed TM3DConvNet using the multi-temporal-phase DCE-MRI.

### 3.3. Spatiotemporal Attention Visualization and Model Interpretation

**Figure 5:**
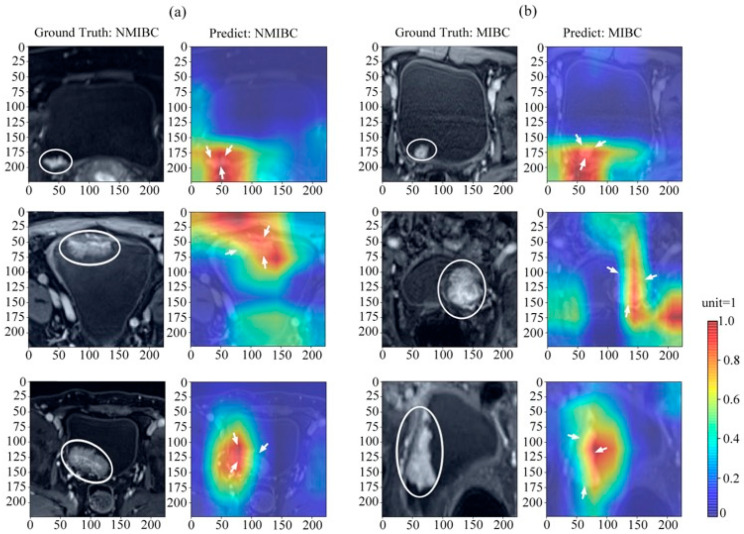
Spatiotemporal attention visualization for (**a**) NMIBC and (**b**) MIBC prediction using multi-temporal-phase DCE-MRI, and white elliptical circules and white arrows represent the tumor region in both CT images and the corresponding hot maps, respectively.

**Figure 6:**
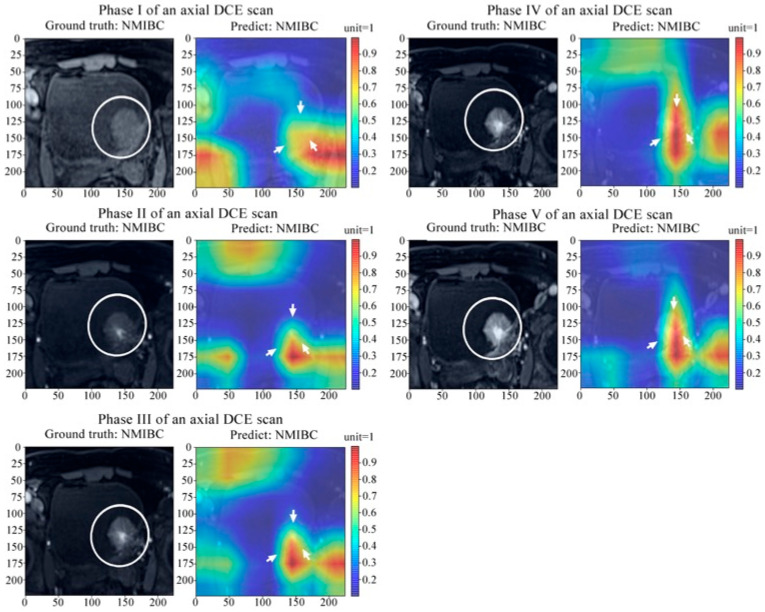
Spatiotemporal attention transition across DCE-MRI phases for non-muscle-invasive bladder cancer characterization, and white elliptical circules and white arrows represent the tumor region in both CT images and the corresponding hot maps, respectively.

To visualize the spatiotemporal attention and attention transition across DCE-MRI phases and interpret the model mechanism, gradient-weighted class activation mapping was applied in feature extraction and classification. Figure 5 shows the spatiotemporal attention visualized for (a) NMIBC and (b) MIBC prediction using multi-temporal-phase DCE-MRI. Figure 6 displays the dynamic distribution characteristics of the attention mechanism in a deep learning network during the inference process of axial Dynamic Contrast-Enhanced Magnetic Resonance Imaging (axial DCE-MRI) across multiple phases (Phase I–V). Each group of subfigures contains two main components: the original DCE-MRI images (left grayscale images with white circles marking the lesion areas) and the corresponding network attention heatmaps (right color images where red and yellow highlight regions of concentrated attention). Additionally, the “Ground truth” (true label) and “Predict” (predicted label) for each phase were both NMIBC (Non-Muscle Invasive Bladder Cancer).

From the visualized results, it can be seen that in different DCE-MRI phases, the network’s attention precisely focused on the areas adjacent to the lesions. At the same time, there were significant differences in the spatial distribution and activation intensity of the attention heatmaps across various phases. This indicates that the network’s attention mechanism is not fixed but dynamically adjusts according to the imaging features of different DCE-MRI phases.

Technical Limitation and Future Directions: Notably, the current 3 mm slice thickness in our DCE-MRI acquisitions (as specified in Figure 5’s caption) presented a fundamental limitation for pixel-level localization of microstructures—particularly the tumor–myometrium boundary—despite our implementation of hybrid 1 × 1 × 1 and 3 × 3 × 3 convolutional kernels. This physical imaging constraint underscores the need for future investigations to integrate: (1) higher-resolution imaging protocols (e.g., 1.5 mm thin-section scanning), and (2) anatomy-guided attention mechanisms to enhance the spatial correspondence between predictive heatmaps and clinically critical features.

In addition, the TM3DConvNet model was further tested on sagittal multi-temporal-phase DCE-MRI data from 5 initially excluded cases, achieving 60% accuracy. Figure S3 presents the spatiotemporal attention visualization for predicting some of these BCa cases with sagittal multi-temporal-phase DCE-MRI data. Figure S4 illustrates the spatiotemporal attention transition across sagittal DCE-MRI phases for one of these cases, specifically for non-muscle-invasive BCa characterization.

## 4. Discussion

DCE-MRI is a valuable imaging modality that tracks the movement of a gadolinium-based contrast agent within tissues and blood vessels over time [12]. This technique enhances signal sensitivity to effectively highlight blood flow, vascular permeability, and the distinct perfusion patterns associated with tumors, thereby providing crucial insights into the pathophysiological and morphological characteristics of tumor masses [12]. For preoperative diagnosis of muscularis invasion in patients with VI-RADS scores of 2 or 3, multi-temporal-phase DCE-MRI is invaluable. This technique, particularly in the early and late phases after contrast administration, emphasizes key features of the bladder’s inner layers, such as the urothelium and lamina propria, which are essential in predicting NMIBC [9].

Although DCE-MRI has unique advantages in visualizing tumor-associated vascular dynamics and the layered structure of the bladder wall, we acknowledge that its inherent low spatial resolution may limit the conclusiveness of image interpretation, particularly when evaluating fine anatomical structures or subtle enhancement patterns. We believe that addressing this issue requires collaborative innovation across imaging, analysis, and clinical practice. This integrated strategy—including techniques such as super-resolution reconstruction, better explainable AI (XAI) methods and clinical validation loop—is not only essential for advancing imaging technology, but also a cornerstone for achieving precise diagnosis and precision therapy.

In this study, we introduced a 3D DL model termed TM3DConvNet, specifically designed to extract the spatiotemporal information inherent in multi-temporal-phase DCE-MRI data for the noninvasive prediction of muscle invasion of BCa patients with VI-RADS scores of 2 or 3. To the best of our knowledge, this is the first investigation of employing multi-temporal-phase DCE-MRI in conjunction with a 3D DL strategy for the diagnosis of NMIBC. Although the number of slices per patient was relatively small, the key slices from the five phases complemented each other in the temporal dimension, providing the model with a complete information chain of tumor dynamic enhancement features. A key component of the proposed model is the MHMC. This module diverges from traditional models that depend on single-scale convolutional kernels arranged across multiple layers. Instead, the MHMC efficiently captures multiscale features from various receptive fields in three-dimensional data using just two convolutional layers. This optimization not only streamlines the model architecture but also minimizes the number of parameters, helping to alleviate computational demands. Our core future work will be to conduct multi-center, large-sample external validation to further consolidate the generalizability of our study conclusions. To further enhance the extraction and fusion of the spatiotemporal attention within these features, we integrated FWAM. Unlike conventional ViT models which focus primarily on global self-attention among input tokens, the FWAM could well fuse both global (coarse) and local (fine-grained) attention, thus enabling a comprehensive synthesis of spatiotemporal information. The ablation studies substantiate that TM3DConvNet, through its dual integration of the MHMC and FWAM modules, significantly outperforms configurations employing either module in isolation.

When assessed against established benchmark networks such as VGG-16, ResNet-101, DenseNet-121 and ViT, TM3DConvNet exhibited superior predictive capabilities for BCa detrusor muscle invasion prediction. This underscores the crucial requirement to capture both multiscale spatial characteristics and temporal dynamics inherent in multi-temporal-phase DCE-MRI data during BCa phenotyping. Furthermore, our findings indicate that the 3D model utilizing comprehensive five-phase DCE-MRI data significantly outperformed their two-dimensional counterparts using single-phase data, solidifying the value of multi-temporal-phase DCE-MRI in BCa diagnosis.

Specifically, when compared to the conventional VI-RADS ≤ 2 threshold. which achieved a sensitivity of 0.67 for NMIBC diagnosis, our proposed TM3DConvNet model demonstrated great performance improvement, with the sensitivity largely improved to 0.90 for NMIBC identification and an AUC of 0.82 for overall classification on the testing cohort.

Using gradient-weighted class activation mapping (Grad CAM), we observed that TM3DConvNet effectively targets the tumor and surrounding bladder wall to extract and classify multiscale features. Notably, our results also reveal important shifts in spatiotemporal attention across the various phases of DCE-MRI, emphasizing the essential role of multiphase imaging in predicting NMIBC.

Nonetheless, it is important to recognize the limitations of this study. The findings are constrained by the reliance on a single-center database, which might impact the generalizability of our results. To enhance clinical applicability, future work should focus on larger, multicenter datasets incorporating diverse MRI platforms. Moreover, the manual cropping of the bladder region using rectangular boxes to exclude complex background has proven to be time-consuming. Future research could further explore fully automated AI segmentation tools (e.g., deep learning models based on U-Net) to completely replace manual operations, thereby further improving efficiency and objectivity. Developing a multitask pipeline that integrates automatic bladder segmentation alongside NMIBC prediction could significantly improve clinical workflow. Finally, considering the potential of mp-MRI in NMIBC diagnostics, future research should explore a static-dynamic integration deep learning model capable of extracting static features from non-contrast sequences, such as T2-weighted MRI, diffusion-weighted MRI, and apparent diffusion coefficient maps, in conjunction with dynamic features from multi-temporal-phase DCE-MRI. This approach could not only advance bladder cancer diagnosis but also benefit other oncological predictions.

## 5. Conclusions

In summary, the proposed TM3DConvNet model demonstrates a robust capacity to extract multiscale spatiotemporal information from multi-temporal-phase DCE-MRI, thereby improving the assessment of detrusor muscularis invasion status among diagnostically challenging BCa cases with VI-RADS 2 or 3.

## Figures and Tables

**Table 1:** Demographics of patients enrolled in this study.

Variables, *n* (%) No. of Patients	Training (*n* = 115)		Validation (*n* = 20)		Testing (*n* = 49)	
	NMIBC (*n* = 86)	MIBC (*n* = 29)	NMIBC (*n* = 13)	MIBC (*n* = 7)	NMIBC (*n* = 37)	MIBC (*n* = 12)
Age						
Median/yr	67.5	65.5	65	66	66	64.5
IQR/yr	[61, 71]	[55, 69]	[57, 70]	[59, 72]	[57, 72]	[58, 71]
Sex, No. (%)						
Female	7 (8.1%)	2 (6.9%)	1 (7.7%)	0	4 (10.8%)	1 (8.3%)
Male	79 (91.9%)	27 (93.1%)	12 (92.3%)	7 (100%)	33 (89.2%)	11 (91.7%)
Smoking, No. (%)						
Never	55 (63.9%)	16 (55.2%)	5 (38.5%)	2 (28.6%)	22 (59.5%)	7 (58.3%)
Ex-/current	31 (36.1%)	13 (44.8%)	8 (61.5%)	5 (71.4%)	15 (40.5%)	5 (41.7%)
Tumors, No. (%)						
<2	68 (79.1%)	23 (79.3%)	9 (69.2%)	4 (57.1%)	28 (75.7%)	9 (75.0%)
2–4	18 (20.9%)	6 (20.7%)	4 (30.8%)	3 (42.9%)	9 (24.3%)	3 (25.0%)
Tumor size						
Median/mm	19	26	22	26	18	25
IQR/mm	[16, 25]	[21, 32]	[16, 30]	[18, 34]	[16, 22]	[19, 36]
Grade, No. (%)						
Low grade	54 (62.8%)	8 (27.6%)	8 (61.5%)	0	21 (56.8%)	3 (25.0%)
High grade	32 (37.2%)	21 (72.4%)	5 (38.5%)	7 (100%)	16 (43.2%)	9 (75.0%)
Operation, No. (%)						
TURBT	76 (88.4%)	3 (10.3%)	9 (69.2%)	1 (14.3%)	31 (83.8%)	1 (8.3%)
Non-TURBT	10 (11.6%)	26 (89.7%)	4 (30.8%)	6 (85.7%)	6 (16.2%)	11 (91.7%)
VI-RADS, No. (%)						
2	70 (81.4%)	8 (27.6%)	9 (69.2%)	1 (14.3%)	28 (75.7%)	4 (33.3%)
3	16 (18.6%)	21 (72.4%)	4 (30.8%)	6 (85.7%)	9 (24.3%)	8 (66.7%)

NMIBC: non-muscle-invasive bladder cancer; MIBC: muscle-invasive bladder cancer; IQR: Interquartile range; TURBT: Transurethral Resection of Bladder Tumor.

**Table 2:** Quantitative comparison of the proposed TM3DConvNet and other deep learning models in NMIBC prediction.

Dataset	Models	SEN	SPE	ACC	AUC
Training cohort	VI-RADS ≤ 2	0.72	0.81	0.79	/
	VGG-16	0.68 ± 0.03	0.78 ± 0.07	0.76 ± 0.08	0.78 ± 0.03
	ResNet-101	0.71 ± 0.08	0.78 ± 0.08	0.77 ± 0.08	0.83 ± 0.07
	DenseNet-121	0.62 ± 0.09	0.83 ± 0.13	0.79 ± 0.08	0.85 ± 0.12
	ViT	0.62 ± 0.11	0.78 ± 0.10	0.75 ± 0.05	0.76 ± 0.13
	**TM3DConvNet**	**0.88 ± 0.06**	**0.83 ± 0.04**	**0.84 ± 0.08**	**0.89 ± 0.03**
Validation cohort	VI-RADS ≤ 2	0.69	0.86	0.75	**/**
	VGG-16	0.67 ± 0.09	**0.80 ± 0.10**	0.73 ± 0.09	0.74 ± 0.10
	ResNet-101	0.82 ± 0.09	0.74 ± 0.06	0.76 ± 0.08	0.80 ± 0.08
	DenseNet-121	0.78 ± 0.10	0.67 ± 0.04	0.72 ± 0.09	0.73 ± 0.08
	ViT	0.78 ± 0.09	0.71 ± 0.08	0.74 ± 0.03	0.75 ± 0.12
	**TM3DConvNet**	**0.87 ± 0.03**	0.78 ± 0.06	**0.79 ± 0.12**	**0.84 ± 0.05**
Testing cohort	VI-RADS ≤ 2	0.67	0.76	0.73	**/**
	VGG-16	0.58 ± 0.11	**0.78 ± 0.07**	0.73 ± 0.03	0.74 ± 0.03
	ResNet-101	0.80 ± 0.09	0.70 ± 0.09	0.73 ± 0.06	0.77 ± 0.04
	DenseNet-121	0.76 ± 0.09	0.68 ± 0.10	0.70 ± 0.06	0.71 ± 0.06
	ViT	0.76 ± 0.11	0.76 ± 0.07	0.76 ± 0.03	0.74 ± 0.03
	**TM3DConvNet**	**0.90 ± 0.10**	0.74 ± 0.07	**0.78 ± 0.05**	**0.82 ± 0.06**

VGG: Visual Geometry Group Network; ViT: Visual Transformer; TM3DConvNet: Transformer-fused multiscale 3D Convolutional network; SEN: Sensitivity; SPE: Specificity; ACC: Accuracy; AUC: Area under the curve.

**Table 3:** Receiver operating characteristic curves comparison of TM3DConvNet and other deep learning models in the testing cohort using DeLong’s tests and Bonferroni correction.

Models	Parameters (Million)	AUC	[95% Confidence Interval]	*p*
VGG-16	512	0.74	[0.71, 0.77]	**<0.05**
ResNet-101	162	0.77	[0.73, 0.81]	0.131
DenseNet-121	27	0.71	[0.65, 0.77]	**<0.05**
ViT	1146.8	0.74	[0.71, 0.77]	**<0.05**
**TM3DConvNet**	**27.9**	**0.82**	**[0.76, 0.88]**	**Ref**

VGG: Visual Geometry Group Network; ViT: Visual Transformer; TM3DConvNet: Transformer-fused multiscale 3D Convolutional network; AUC: Area under the curve, *p* < 0.05 is considered statistically significant. Ref indicates that this model was used as the reference to compare with other models.

**Table 4:** Ablation experiments on primary modules for NMIBC prediction in the testing cohort.

MHMC	FWAM	SEN	SPE	ACC	AUC
√	×	0.62 ± 0.07	0.75 ± 0.07	0.72 ± 0.05	0.70 ± 0.10
×	√	0.77 ±0.10	0.75 ± 0.07	0.76 ± 0.05	0.75 ± 0.05
**√**	**√**	**0.90 ± 0.10**	0.74 ± 0.07	**0.78 ± 0.05**	**0.82 ± 0.06**

MHMC: Multi-head mixed 3D convolution; FWAM: Fusion Window Attention module; SEN: Sensitivity; SPE: Specificity; ACC: Accuracy; AUC: Area under the curve.

## Data Availability

The original contributions presented in the study are included in the article/Supplementary Material, further inquiries can be directed to the corresponding author.

## Supplementary Materials

The following supporting information can be downloaded at: https://www.mdpi.com/article/10.3390/bioengineering12121338/s1, Figure S1: Inclusion and exclusion criteria of the present study; Figure S2: Loss curves of the proposed TM3DconvNet and benchmark models in model training process for detrusor muscularis invasion prediction; Figure S3: Spatiotemporal attention visualization for predicting (a) NMIBC and (b–d) MIBC cases with sagittal multiphase DCE-MRI data; Figure S4: Spatiotemporal attention transition across sagittal DCE-MRI phases for NMIBC characterization; Figure S5: Images generated by conditional generative adversarial networks; Figure S6: MHMC Module Code Example; Figure S7: FWAM Module Code Example; Table S1: Predominant scanning parameters of multiparametric MRI; Table S2: Definition and Time Points of Multi-temporal-phase DCE-MRI; Table S3: Testing performance of the corresponding deep models with one of the five phases DCE-MRI in NMIBC prediction.

## Abbreviations

The following abbreviations are used in this manuscript:

BCa	Bladder Cancer
MRI	Magnetic Resonance Imaging
DCE-MRI	Dynamic Contrast-Enhanced Magnetic Resonance Imaging
NMIBC	Non-Muscle-Invasive Bladder Cancer
MIBC	Muscle-Invasive Bladder Cancer
AUC	Area Under the Curve
T2WI	T2-Weighted Imaging
DWI	Diffusion-Weighted Imaging
VI-RADS	Vesical Imaging-Reporting and Data System
TM3DConvNet	Transformer-fused Multiscale 3D Convolutional Network
MHMC	Multi-Head Mixed Convolution
FWAM	Fusion Window Attention Module

## References

[1] Bray F., Laversanne M., Sung H., Ferlay J., Siegel R.L., Soerjomataram I., Jemal A. (2024). Global cancer statistics 2022: GLOBOCAN estimates of incidence and mortality worldwide for 36 cancers in 185 countries. CA Cancer J. Clin..

[2] Arita Y., Kwee T.C., Akin O., Shigeta K., Paudyal R., Roest C., Ueda R., Lema-Dopico A., Nalavenkata S., Ruby L. (2025). Multiparametric MRI and artificial intelligence in predicting and monitoring treatment response in bladder cancer. Insights Imaging.

[3] Gontero P., Birtle A., Capoun O., Compérat E., Dominguez-Escrig J.L., Liedberg F., Mariappan P., Masson-Lecomte A., Mostafid H.A., Pradere B. (2024). European Association of Urology Guidelines on Non-muscle-invasive Bladder Cancer (TaT1 and Carcinoma In Situ)-A Summary of the 2024 Guidelines Update. Eur. Urol..

[4] Liu Y., Xu X., Wang H., Liu Y., Wang Y., Dong Q., Li Z., Guo Y., Lu H. (2023). The Additional Value of Tri-parametric MRI in Identifying Muscle-invasive Status in Bladder Cancer. Acad. Radiol..

[5] Witjes J.A., Bruins H.M., Carrión A., Cathomas R., Compérat E., Efstathiou J.A., Fietkau R., Gakis G., Lorch A., Martini A. (2024). European Association of Urology Guidelines on Muscle-invasive and Metastatic Bladder Cancer: Summary of the 2023 Guidelines. Eur. Urol..

[6] Cai L., Yu R., Liu P., Zhuang J., Li K., Wu Q., Sun X., Liu Y., Zhou M., Cao Q. (2024). A Nomogram of MRI Features to Assess Muscle Invasion in VI-RADS 2 Tumors with Stalk. J. Magn. Reson. Imaging.

[7] Yu R., Cai L., Cao Q., Liu P., Gong Y., Li K., Wu Q., Zhang Y., Li P., Yang X. (2024). Development and Validation of an MRI-Based Nomogram for Preoperative Detection of Muscle Invasion in VI-RADS 3. J. Magn. Reson. Imaging.

[8] Xu X., Huang Y., Liu Y., Cai Q., Guo Y., Wang H., Lu H. (2024). Multiparametric MRI-based VI-RADS: Can it predict 1- to 5-year recurrence of bladder cancer?. Eur. Radiol..

[9] Huang H., Huang Y., Kaggie J.D., Cai Q., Yang P., Wei J., Wang L., Guo Y., Lu H., Wang H. (2025). Multiparametric MRI-Based Deep Learning Radiomics Model for Assessing 5-Year Recurrence Risk in Non-Muscle Invasive Bladder Cancer. J. Magn. Reson. Imaging.

[10] Panebianco V., Narumi Y., Altun E., Bochner B.H., Efstathiou J.A., Hafeez S., Huddart R., Kennish S., Lerner S., Montironi R. (2018). Multiparametric Magnetic Resonance Imaging for Bladder Cancer: Development of VI-RADS (Vesical Imaging-Reporting and Data System). Eur. Urol..

[11] Nia I.Y., Ambale-Venkatesh B. (2025). Editorial for “Multiparametric MRI-Based Deep Learning Radiomics Model for Assessing 5-Year Recurrence Risk in Non-Muscle Invasive Bladder Cancer”. J. Magn. Reson. Imaging.

[12] Ueno Y., Takeuchi M., Tamada T., Sofue K., Takahashi S., Kamishima Y., Hinata N., Harada K., Fujisawa M., Murakami T. (2019). Diagnostic Accuracy and Interobserver Agreement for the Vesical Imaging-Reporting and Data System for Muscle-invasive Bladder Cancer: A Multireader Validation Study. Eur. Urol..

[13] Wang H., Luo C., Zhang F., Guan J., Li S., Yao H., Chen J., Luo J., Chen L., Guo Y. (2019). Multiparametric MRI for Bladder Cancer: Validation of VI-RADS for the Detection of Detrusor Muscle Invasion. Radiology.

[14] Schaff L.R., Mellinghoff I.K. (2023). Glioblastoma and Other Primary Brain Malignancies in Adults. JAMA.

[15] Welk B., McArthur E., Morrow S.A., Macdonald P., Hayward J., Leung A., Lum A. (2016). Association Between Gadolinium Contrast Exposure and the Risk of Parkinsonism. JAMA.

[16] Huang H., Mo J., Ding Z., Peng X., Liu R., Zhuang D., Zhang Y., Hu G., Huang B., Qiu Y. (2025). Deep Learning to Simulate Contrast-Enhanced MRI for Evaluating Suspected Prostate Cancer. Radiology.

[17] Elshetry A.S.F., El-Fawakry R.M., Hamed E.M., Metwally M.I., Zaid N.A. (2022). Diagnostic accuracy and discriminative power of biparametric versus multiparametric MRI in predicting muscle-invasive bladder cancer. Eur. J. Radiol..

[18] Pizzi A.D., Mastrodicasa D., Marchioni M., Primiceri G., Di Fabio F., Cianci R., Seccia B., Mincuzzi E., Romanelli M., Castellan P. (2020). Bladder cancer: Do we need contrast injection for MRI assessment of muscle invasion? A prospective multi-reader VI-RADS approach. Eur. Radiol..

[19] Zhang L., Li X., Yang L., Tang Y., Guo J., Li D., Li S., Li Y., Wang L., Lei Y. (2023). Multi-Sequence and Multi-Regional MRI-Based Radiomics Nomogram for the Preoperative Assessment of Muscle Invasion in Bladder Cancer. J. Magn. Reson. Imaging.

[20] Xu C., Xu L., Ohorodnyk P., Roth M., Chen B., Li S. (2020). Contrast agent-free synthesis and segmentation of ischemic heart disease images using progressive sequential causal GANs. Med. Image Anal..

[21] Yu Y., Chen R., Yi J., Huang K., Yu X., Zhang J., Song C. (2024). Non-invasive prediction of axillary lymph node dissection exemption in breast cancer patients post-neoadjuvant therapy: A radiomics and deep learning analysis on longitudinal DCE-MRI data. Breast.

[22] Zhou J., Zhang Y., Chang K., Lee K.E., Wang O., Li J., Lin Y., Pan Z., Chang P., Chow D. (2019). Diagnosis of Benign and Malignant Breast Lesions on DCE-MRI by Using Radiomics and Deep Learning with Consideration of Peritumor Tissue. J. Magn. Reson. Imaging.

[23] Li M., Fan Y., You H., Li C., Luo M., Zhou J., Li A., Zhang L., Yu X., Deng W. (2023). Dual-Energy CT Deep Learning Radiomics to Predict Macrotrabecular-Massive Hepatocellular Carcinoma. Radiology.

[24] Witowski J., Heacock L., Reig B., Kang S.K., Lewin A., Pysarenko K., Patel S., Samreen N., Rudnicki W., Łuczyńska E. (2022). Improving breast cancer diagnostics with deep learning for MRI. Sci. Transl. Med..

[25] Bedel H.A., Sivgin I., Dalmaz O., Dar S.U., Çukur T. (2023). BolT: Fused window transformers for fMRI time series analysis. Med. Image Anal..

[26] Shen L., Sun M., Li Q., Li B., Pan Z., Lei J. (2022). Multiscale Temporal Self-Attention and Dynamical Graph Convolution Hybrid Network for EEG-Based Stereogram Recognition. IEEE Trans. Neural Syst. Rehabil. Eng..

[27] Xia L., Xie Y., Wang Q., Zhang H., He C., Yang X., Lin J., Song R., Liu J., Zhao Y. (2021). A nested parallel multiscale convolution for cerebrovascular segmentation. Med. Phys..

[28] Zadeh F.S., Pooyan A., Alipour E., Hosseini N., Thurlow P.C., Del Grande F., Shafiei M., Chalian M. (2024). Dynamic contrast-enhanced magnetic resonance imaging (DCE-MRI) in differentiation of soft tissue sarcoma from benign lesions: A systematic review of literature. Skelet. Radiol..

[29] Wang H., Xu X., Zhang X., Liu Y., Ouyang L., Du P., Li S., Tian Q., Ling J., Guo Y. (2020). Elaboration of a multisequence MRI-based radiomics signature for the preoperative prediction of the muscle-invasive status of bladder cancer: A double-center study. Eur. Radiol..

[30] Isola P., Zhu J.-Y., Zhou T., Efros A.A. Image-to-Image Translation with Conditional Adversarial Networks. Proceedings of the IEEE Conference on Computer Vision & Pattern Recognition.

[31] You S., Masutani E.M., Alley M.T., Vasanawala S.S., Taub P.R., Liau J., Roberts A.C., Hsiao A. (2022). Deep Learning Automated Background Phase Error Correction for Abdominopelvic 4D Flow MRI. Radiology.

