# Supplementary material for: Beyond VI-RADS Uncertainty: Leveraging Spatiotemporal DCE-MRI to Predict Bladder Cancer Muscle Invasion
Source: Bioengineering (Basel). 2025 Dec 8;12(12):1338. doi: 10.3390/bioengineering12121338 (PMC12729362; doi:10.3390/bioengineering12121338)
Supplement: Supplementary file 1 [file bioengineering-12-01338-s001.zip › bioengineering-3905821-supplementary.pdf]

Supplementary Materials

Beyond VI-RADS Uncertainty: Leveraging Spatiotemporal DCE-MRI to Predict Bladder Cancer Muscle Invasion

Contents

1. *Predominant scanning parameters of multiparametric MRI*................................. *3*

2. *Conditional Generative Adversarial Network*………………………………………………*4*

3. *Multi-Head Mixed 3D Convolution (MHMC) Module* *5*

4. *Transformer-based Fusion Windows Attention Module (FWAM)*.......................... *7*

5. *Classification Module*... *9*

6. *Testing Performance of of the corresponding deep models with one of the five phases DCE-MRI in NMIBC prediction* *9*

7. *Spatiotemporal attention visualization for predicting non-muscle-invasive bladder cancer and NMIBC cases with sagittal multiphase DCE-MRI data*...... *11*

8. *Spatiotemporal attention transition across sagittal DCE-MRI phases for non-muscle-invasive bladder cancer characterization* *12*

9. *Images generated by conditional generative adversarial networks* ………………*12*

10. *Code Example*……………………………………………………………………………….*13*

1. Predominant scanning parameters of multiparametric MRI

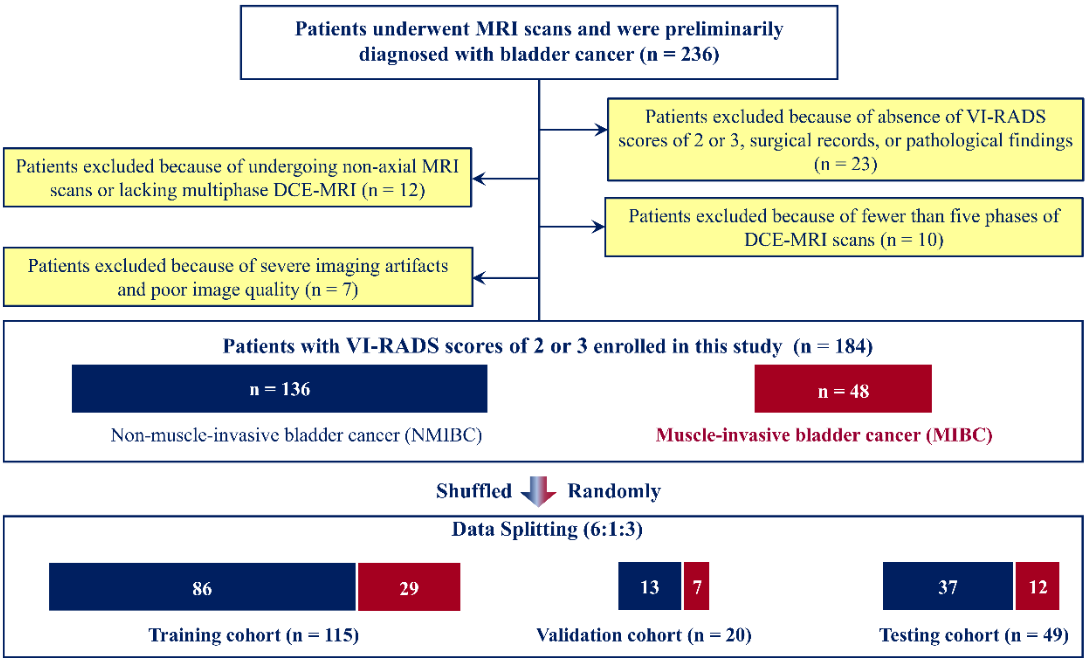

Figure S1 Inclusion and exclusion criteria of the present study

Table S1: Predominant scanning parameters of multiparametric MRI

**Parameters**	**T2W**	**DW**	**DCE**
Sequence	Fast Spin echo	Single-shot EPI	Gradient echo
TR (msec)	3500–4650	400	3.31
TE (msec)	95–102	78	1.22
Flip angle (°)	150	90	13
FOV (mm)	220×220	260×221	300×264
Matrix	320×288	160×112	256×192
Slice thickness (mm)	4	4	2
Slice gap (mm)	0.4	0.4	0.2
Number of excitations	3	8	1
Echo train length	15	-	-
b values	-	0,1000s/mm^2	-

DW = diffusion-weighted; **DCE** = dynamic contrast enhancement; **TR** = repetition time; **TE** = echo time; **FOV** = field of view; **T2W** = T2-weighted

Table S2: Definition and Time Points of Multi-temporal-phase DCE - MRI

Phase Number	Clinical Name	Time Point	Core Function
1	Plain Scan Phase (Baseline)	0 seconds (Before injection)	Provide baseline signal reference for tumors
2	Early Arterial Phase	28~32 seconds	Capture the arterial blood supply enhancement characteristics of tumors
3	Intermediate Phase (Transitional)	55~65 seconds	Assist in observing the transitional enhancement characteristics
4	Venous Phase	85~95 seconds	Reflect the venous return of contrast agent and the relationship between tumor and vein
5	Delayed Phase	5 minutes	Evaluate the retention/clearance of contrast agent (key for myometrial invasion)

2. Conditional Generative Adversarial Network

Conditional Generative Adversarial Network (CGAN) for Targeted Augmentation (Addressing Class Imbalance):

To address the limited number of MIBC samples in the training set (only 35%), we further employed CGAN to generate synthetic MIBC samples for balancing the class distribution. The generator of the CGAN was based on a U-Net architecture, taking a random noise vector (100-dimensional) and a class label (MIBC=1) as inputs. Through five layers of transposed convolutions, it progressively upsampled to produce images (128×128×3, corresponding to the three contrast sequences of DCE-MRI) that matched the size of real MIBC slices. The discriminator adopted a PatchGAN structure, which took real/synthetic images along with their labels as inputs and extracted local features through four layers of convolutions to determine authenticity. During training, the CGAN used real MIBC samples as supervisory signals, minimizing both the adversarial loss and the class conditional loss (to ensure that the synthetic images belonged to the MIBC category). This process generated synthetic images with clinically representative tumor enhancement patterns (such as high signal intensity in

the early arterial phase and contrast agent retention in the delayed phase). Ultimately, 40 synthetic MIBC samples were created (accounting for 30% of the original MIBC samples) and combined with real samples for model training.

3. Multi-Head Mixed 3D Convolution (MHMC) Module

In this study, we introduce a lightweight feature mixer, termed the Multi-Head Mixed 3D Convolution (MHMC) module, to extract multi-scale spatial features from bladder cancer DCE-MRI images. The MHMC module is designed to capture both local and global contextual information by leveraging convolutional kernels of varying sizes.

Input Preprocessing

Each subject's DCE-MRI data consists of five image slices (phases) after preprocessing, denoted as $\boldsymbol{X} \in \boldsymbol{R}^{\boldsymbol{H}\times\boldsymbol{W}\times\boldsymbol{5}}$, where H×W represents the spatial dimensions of a single image slice. These preprocessed images are fed into the MHMC module for feature extraction.

Module Architecture

The MHMC module comprises three parallel dual-layer convolutional branches, each designed to capture features at different spatial scales. Each branch combines traditional 3D convolutional kernels with depthwise separable convolutional kernels to efficiently extract spatial information from the input images. Specifically, the convolutional kernel sizes are chosen to capture features at varying receptive fields:

1×1×1 Kernel: Captures local features with a small receptive field.

3×3×3 Kernel: Captures medium-range features with a moderate receptive field.

5×5×5 Kernel: Captures larger-range features with a broad receptive field.

The feature extraction process in each branch can be formally expressed as:

$$X_i'' = reshape\big(w_b(w_a X)\big), i = 1,3,5$$

where :

◆ X is the input 5-phase image slice with dimensions $H \times W \times 5$.

- w_a and w_b are learnable convolutional kernels in the dual-layer structure.
- reshape $(\cdot)$ is an operation that transforms the feature dimensions from 3D to 2D for further processing.

Feature Concatenation

After extracting features from the three branches, the spatial features are concatenated along the temporal dimension to generate the input tokens $\boldsymbol{X}''$. This concatenation process can be represented as:

$$X'' = Concat({X_1}'', {X_3}'', {X_5}'')$$

The resulting input token $\boldsymbol{X}''$ has dimensions $1 \times 448 \times 784$, encapsulates multi-scale spatial information, enabling the model to capture a broader range of contextual features and long-range dependencies. These tokens serve as the input to the subsequent fused window attention module for further analysis.

Depth-wise Separable Convolutions

To further enhance feature extraction efficiency, the MHMC module employs depth-wise separable convolutions in the second layer of each branch. This approach reduces computational complexity while maintaining the ability to capture multi-scale spatial information. Depth-wise separable convolutions decompose the standard 3D convolution into two steps:

Depth-wise Convolution: Applies a single convolutional filter per input channel.

Pointwise Convolution: Combines the outputs of the depthwise convolution using $1 \times 1 \times 1$ convolutions.

This decomposition significantly reduces the number of parameters and computational cost, making the MHMC module both lightweight and effective.

Output Features

The output features $\boldsymbol{X}''$ generated by the MHMC module are rich in multi-scale spatial information, capturing both fine-grained details and broader contextual patterns. These features are essential for the model to analyze the spatiotemporal dynamics of bladder cancer in DCE-MRI images effectively.

4.Transformer-based Fusion Windows Attention Module (FWAM)

The FWAM consists of a series of modified Transformer blocks and utilizes parallel convolutional structures to extract high-level spatial features. During the computation of local attention, the entire set of time points is initially divided into distinct windows. Next, within each window, a combination of the auxiliary token and the local time series is merged and fed into the block. The auxiliary token is then updated based on the significance of subtle variations in the tumor imaging process. Ultimately, the output auxiliary tokens are aggregated into label embeddings, and a fully connected layer is applied for classification.

Fusion window attention

This module combines shifted window interaction and general multi-head attention mechanisms to compute local correlations between the input auxiliary token and base token within each window. Specifically, the original vector is divided into $F = (T - W) / S + 1$ windows (i.e., F base tokens), where $T = 448$ is the total number of time points, W represents the size of the time points in each window, and the stride between adjacent windows is S. Additionally, the auxiliary token is appended to both sides of the base token to enhance the receptive field and capture detailed temporal information.

Since all windows are processed in a similar manner, we illustrate the attention calculation process using a single window. For the $i - th$ window, the query (Q), key (K), and value (V) vectors are constructed from the main-auxiliary vector pair. Specifically, we directly set the time series $x_i \in R^{W \times N}$ (where N=784 is the encoding dimension) within the i-th window as the main token. The auxiliary tokens ($a_i, b_i \in R^{L \times N}$, where $N = 784$ is the encoding dimension, L denotes the number of fringe tokens on each side of the base token) are derived from the time series adjacent to the main token on both sides. These auxiliary tokens are randomly initialized and correspond to each window.

On the basic of those tokens, the query, key and value vectors are defined as follow:

$$Q_i = W_q\{c_i, x_i\} \in R^{(1+W)\times N}$$

$$K_i = V_i = W_k\{c_i, a_i, x_i, b_i\} \in R^{(1+W+2L)\times N}$$

where W_q and W_k are the learnable projection parameters, $c_i$is a context vector used to enhance the representation of the main token, and the local attention is denoted as:

$$attn(Q_i, K_i, V_i) = \sigma(\frac{Q_i {K_i}^T}{\sqrt{d_i}})V_i$$

where d_i is the dimension of input features, and used to stabilize the gradients during training, σ is $softmax(\cdot)$ function.

Token Fusion

After obtaining the final classification variable C', we aggregate the learned time features from all windows, weighted by their respective importance,

$$F_o = \frac{1}{F}\sum_{i=1}^{F} c_i$$

where F_o is the representative features after feature fusion. Subsequently, layer normalization was applied to stabilize the hidden states at each time point and mitigate the gradient explosion problem.

5. Classification Module

After feature normalization and temporal averaging, these output classification tokens are transformed into an embedding vector that encapsulates both spatial and temporal information. Subsequently, a fully connected layer is employed to predict the label embedding probability y_p for each subject:

$$y_p = \sigma(\boldsymbol{W_o} F_o + b_o)$$

where $\boldsymbol{W_o}$ and b_o are the weight matrix and bias matrix, respectively, and $\sigma()$ is the sigmoid function. Finally, a cross-entropy function is introduced to compute the loss L_{loss} between the true label y and the predicted label y_p, followed by a binary classification decision:

$$L_{loss} = -\frac{1}{N}\sum_{i=1}^{N}[y \cdot \log(y_p) + (1-y) \cdot \log(1-y_p)].$$

6. Performance of of the corresponding deep models with one of the five phases DCE-MRI in NMIBC prediction

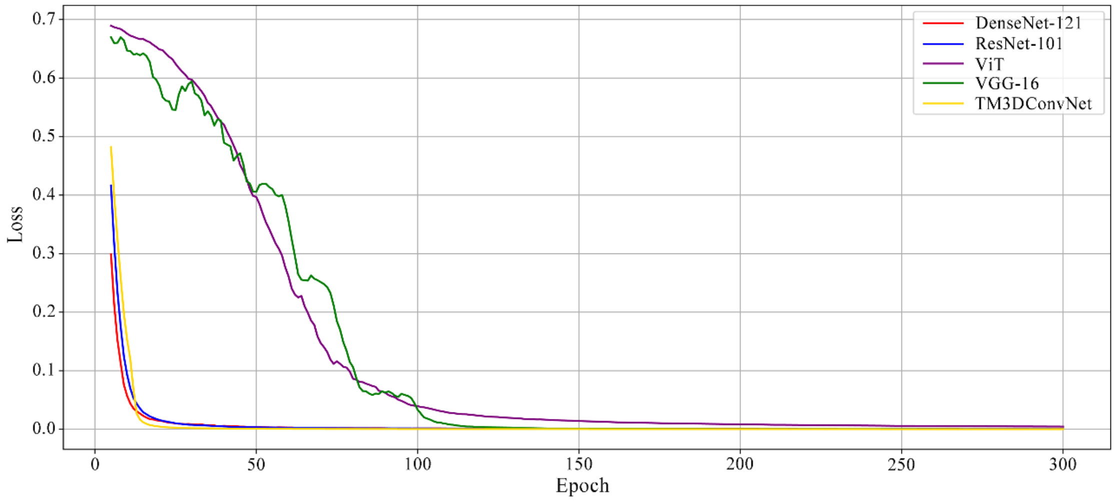

Figure S2 Loss curves of the proposed TM3DconvNet and benchmark models in model training process for detrusor muscularis invasion prediction.

Table S2. Testing Performance of of the corresponding deep models with one of the five phases DCE-MRI in NMIBC prediction

Models	Phase	SEN	SPE	ACC	AUC
VGG-16	1	0.65±0.18	0.63± 0.18	0.67±0.09	0.64±0.04
	2	0.74±0.14	0.63± 0.16	0.66±0.08	0.68±0.01
	3	0.67±0.14	**0.76±0.15**	**0.74±0.08**	0.71±0.02
	4	**0.78±0.08**	0.62±0.09	0.66±0.05	**0.71±0.01**
	5	0.76±0.12	0.66±0.14	0.69±0.07	0.71±0.03
ResNet-101	1	0.78±0.17	0.60±0.16	0.65±0.08	0.68±0.04
	2	0.76±0.09	0.70±0.11	0.71±0.07	0.71±0.03
	3	0.79±0.08	0.63±0.10	0.67±0.05	0.73±0.03
	4	0.72±0.13	**0.72±0.11**	0.72±0.05	0.70±0.02
	5	**0.80±0.09**	0.70±0.09	**0.73±0.06**	**0.76±0.03**
DenseNet-121	1	0.73±0.18	0.66±0.18	0.68±0.09	**0.69±0.05**
	2	0.73±0.19	0.66±0.17	0.68±0.09	0.68±0.06
	3	0.64±0.17	**0.74±0.15**	**0.71±0.08**	0.68±0.05
	4	**0.76±0.13**	0.65±0.12	0.68±0.07	0.68±0.03
	5	0.72±0.15	0.64±0.14	0.66±0.07	0.66±0.05
ViT	1	0.78±0.17	0.60±0.16	0.65±0.08	0.68±0.04
	2	0.76±0.09	0.70±0.11	0.71±0.07	0.71±0.03
	3	**0.79±0.08**	0.63±0.10	0.67±0.05	**0.73±0.03**
	4	0.71±0.13	0.72±0.11	**0.72±0.05**	0.70±0.02
	5	0.72±0.10	**0.73±0.13**	0.72±0.07	0.71±0.06

VGG: Visual Geometry Group Network; ViT: Visual Transformer； SEN: Sensitivity; SPE: Specificity; ACC: Accuracy; AUC: Area under the curve.

7. Spatiotemporal attention visualization for predicting NMIBC and

MIBC cases with sagittal multiphase DCE-MRI data

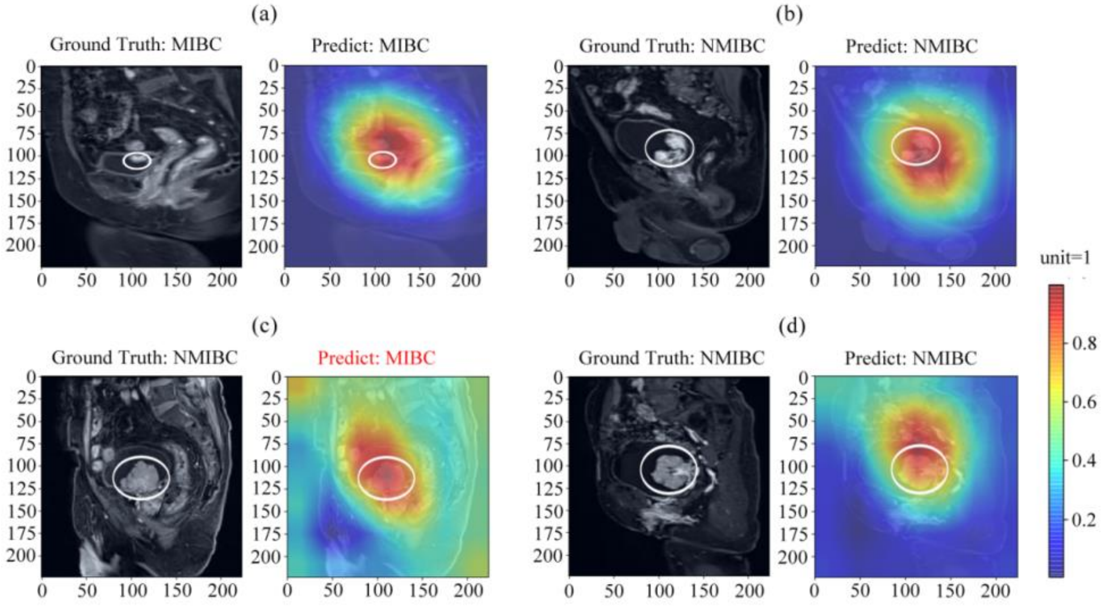

Figure S3 Spatiotemporal attention visualization for predicting (a) NMIBC and (b) - (d) MIBC cases with sagittal multiphase DCE-MRI data

8. Spatiotemporal attention transition across sagittal DCE-MRI phases for NMIBC characterization

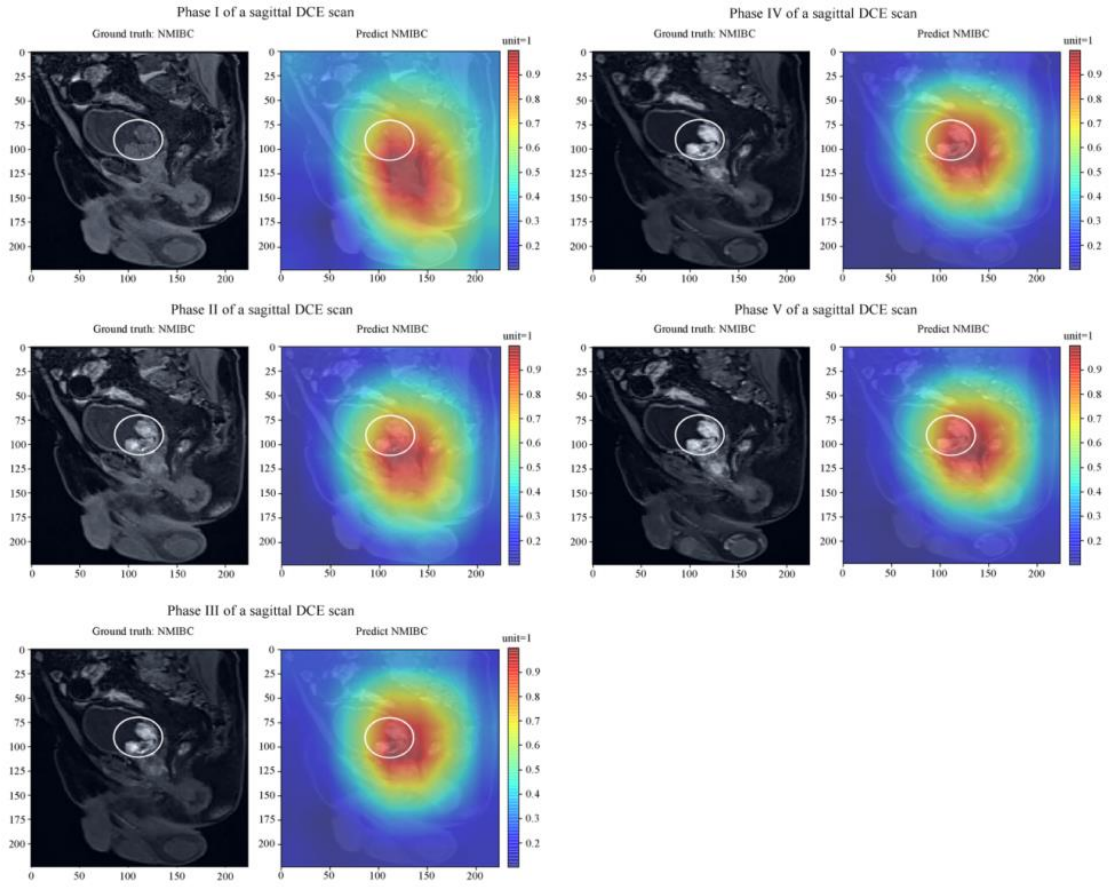

Figure S4 Spatiotemporal attention transition across sagittal DCE-MRI phases for NMIBC characterization

9. Images generated by conditional generative adversarial networks

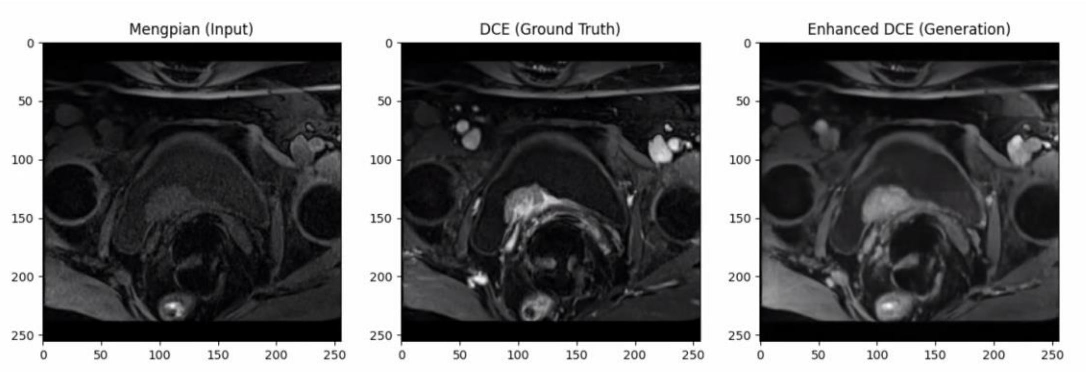

Figure S5 Images generated by conditional generative adversarial networks

10. Code Example

```
class MHMC(nn.Module):
  def __init__(self):
    super().__init__()
    # Branch1: 1x1x1 conv (Fine)
    self.branch1 = nn.Sequential(
      # Suppose input is composed of single-channel slices with 5 time points
      nn.Conv3d(1, 16, kernel_size=(1,1,1), padding=0),
      nn.Conv3d(16, 32, kernel_size=(1, 1, 1), groups=32) # Depthwise separable convolution
    )
    # Branch2: 3x3x3 conv (Region)
    self.branch3 = nn.Sequential(
      nn.Conv3d(1, 16, kernel_size=(3, 3, 3), padding=1),
      nn.Conv3d(16, 32, kernel_size=(1, 1, 1), groups=32)
    )
    # Branch3: 5x5x5 conv (Coarse)
    self.branch5 = nn.Sequential(
      nn.Conv3d(1, 16, kernel_size=(5, 5, 5), padding=2),
      nn.Conv3d(16, 32, kernel_size=(1, 1, 1), groups=32)
    )
    self.reshape = nn.Flatten(start_dim=1)  # Conversion from 3D to 2D
```

Figure S6 MHMC Module Code Example

```
class FWAM(nn.Module):
  def __init__(self):
    super().__init__()
    self.W_q = nn.Linear((1+W)*N, q_dim) # query mapping
    self.W_k = nn.Linear((1+W+2*L)*N, kv_dim) # key & value mapping

  def forward(self, x_i, a_i, b_i, c_i):
    # Build the query, key, and value (Refer to the formula in the paper)
    Q_i = self.W_q(torch.cat([c_i, x_i], dim=-1))   # Q_i = W_q{c_i, x_i}
    K_i = V_i= self.W_k(torch.cat([c_i, a_i, x_i, b_i], dim=-1))   # K_i = V_i = W_k{c_i, a_i, x_i, b_i}
    attn_out = F.softmax(torch.matmul(Q_i, K_i.T)/torch.sqrt(d_i), dim=-1) @ V_i  # Local
attention
    return attn_out
```

Figure S7 FWAM Module Code Example
